# A Novel Sodium–Potassium Anode Supported by Fluorinated Aluminum Foam

**DOI:** 10.3390/ma16237269

**Published:** 2023-11-22

**Authors:** Jin Lou, Jingan Zhou, Xiaosong Ma, Kanghua Chen, Songyi Chen

**Affiliations:** 1 Light Alloy Metal Research Institute, Central South University, Changsha 410083, China; khchen@csu.edu.cn (K.C.); sychen08@csu.edu.cn (S.C.); 2 Hunan Provincial Key Laboratory of Carbon Neutrality and Intelligent Energy, School of Resources & Environment, Hunan University of Technology and Business, Changsha 410205, China; 17373564507@163.com (J.Z.); ma15367552578@163.com (X.M.)

**Keywords:** restrain dendrite, sodium–potassium alloy, aluminum foam, cycle stability

## Abstract

Sodium–potassium (NaK) liquid alloy is a promising candidate for use as an anode material in sodium batteries because of its fluidity, which effectively suppresses the growth of sodium or potassium dendrites. However, the poor wettability of NaK alloy on conventional metal substrates is unfavorable for cell fabrication due to its strong surface tension. In this paper, low-density and low-cost fluorinated aluminum foam is used as a substrate support material for NaK liquid alloy. By combining low-surface-tension NaKC with fluorinated aluminum foam, we obtain a uniformly distributed and structurally stable electrode material. The composite electrode has a cycling stability of more than 3000 h in a symmetrical cell. Furthermore, when coupled with a sulfurized polyacrylonitrile cathode in carbonate electrolyte, it maintains excellent stability even after 800 cycles, with 72% of capacity retention.

## 1. Introduction

Sodium–potassium (Na-K) alloy is a liquid-phase alloy that remains in a liquid state at room temperature, and the abundance of sodium and potassium elements is over a thousand times greater than that of lithium [1,2]. As a liquid-phase anode, Na-K alloy is a promising approach to realize dendrite-free stripping/deposition of alkali metals, and it has been reported as a new type of anode material in sodium or potassium batteries that can inhibit Na or K dendrite growth [3,4]. Due to the fluidity of Na-K alloy, it is difficult to form a stable electrode. Therefore, it is necessary to compound Na-K alloy with porous material so as to realize the fixation of the liquid alloy.

Porous metal skeletons, such as foam copper, foam nickel and foam aluminum, are known for their good conductivity and ability to evenly distribute the metal-ion flux through spatial segmentation, which can effectively suppress dendrite growth [5,6,7,8]. Among them, foam aluminum is highly potential as an electrochemical substrate material due to its light weight and high abundance, and it has been used in research on deposition with sodium metal [9,10,11]. However, due to the high surface tension of Na-K, it is difficult to form stable and homogeneous electrode materials with a typical porous metal skeleton [12]. Therefore, to obtain a stable Na-K liquid alloy electrode, the imperative strategy is to reduce the surface tension of Na-K liquid alloy and enhance the affinity of the metal skeleton to Na-K liquid metal.

In this study, graphite powder was added to sodium–potassium alloy, causing a pre-reaction that reduced the surface tension of the liquid alloy. Then, the Na-K liquid alloy was fully adsorbed in fluorinated foam aluminum via vacuum processing, forming an NaKC-FAF composite electrode. This composite anode material was tested in a symmetrical cell and a full battery, and it demonstrated outstanding cycling performance.

## 2. Experiments

### 2.1. Preparation of the Fluorinated Foam Aluminum

The foam aluminum was immersed in an aqueous solution containing 0.1% hydrogen fluoride for 10 to 20 s. It was then quickly transferred to a dimethyl carbonate (DMC) solvent to remove excess hydrogen fluoride solution. After that, it was dried for 2 h in a vacuum atmosphere for the next step.

### 2.2. Preparation of NaKC-FAF Electrode

To prepare liquid sodium–potassium alloy, solid metallic potassium and sodium were mechanically stirred (at a mass ratio of 1:1) with a glass rod in an argon-filled glovebox. After stirring to form the liquid alloy, 0.2 g of graphite powder (GP) was added to 1 g liquid alloy and stirred for another 15 min to reduce the surface tension of the liquid alloy. The resulting composite was named NaKC. NaKC was mixed with fluorinated foam aluminum, ensuring that the foam aluminum (AF) was completely immersed in the liquid alloy. The mixture was put into a transition chamber for repeated vacuum operations over three times to obtain NaKC-FAF electrode.

### 2.3. Preparation of SPAN Electrode and Batteries Assembly

For the cathode, sublimed sulfur was mixed with polyacrylonitrile (PAN) at a mass ratio of 4:1. The mixture was then heated at 350 °C and aged for 6 h in flowing inert gas, resulting in a black, powdery substance called SPAN (sulfur content was 40%). The cathode was fabricated by mixing 70 wt% SPAN, 20 wt% acetylene black and 10 wt% guar gum. The loading amount of SPAN cathode was about 1 mg cm^−2^_sulfur_. CR2032-type cells were assembled separately with Na foil and NaKC-FAF as the anode, Canrd 3501 (Dongguan, China) as the separator, and a 1.0 mol/L NaClO_4_ solution in a mixture of ethylene carbonate (EC) and propylene carbonate (PC) (1:1 wt.%) with 5% fluoroethylene carbonate (FEC) as the electrolyte. Additionally, the electrolyte-to-sulfur ratio was 30 ul/g_span_.

### 2.4. Electrochemical Testing and Characterization

Electrochemical performance was tested using a LAND-CT2001A cycler (Wuhan, China) under ambient conditions. The chemical component and morphologies of the electrodes were determined using X-ray Photoelectron Spectroscopy (XPS, Axis-UltraDLD, Kratos Analytical, Hadano, Japan) and a scanning electron microscope (SEM, Quanta FEG 250, FEI, Changsha, China).

## 3. Results and Discussion

**Figure 1:**
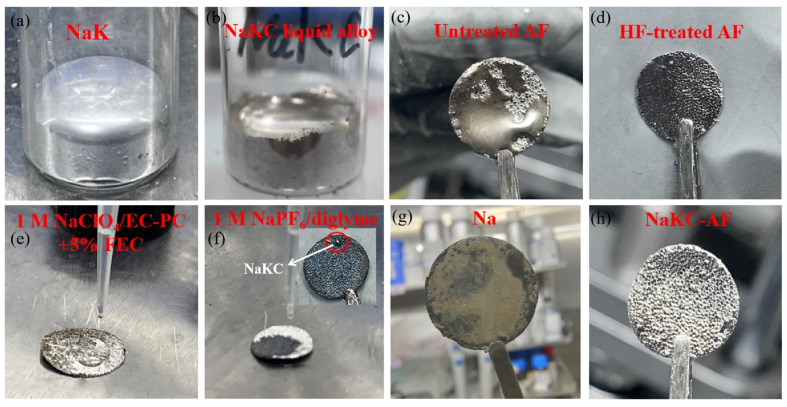
Optical photographs of (**a**) NaK liquid alloy, (**b**) graphite powder added to NaK liquid alloy, (**c**) NaKC on AF (without HF treatment), (**d**) NaKC-FAF, (**e**,**f**) different electrolytes dropped onto NaKC-FAF, and (**g**,**h**) cycled Na anode and NaKC-FAF anode.

The liquid surface of NaK in Figure 1a exhibits a convex phenomenon, while in Figure 1b, NaKC presents a concave phenomenon. It could be concluded that the prepared NaK liquid alloy showed a high surface tension, while the surface tension of NaKC noticeably decreased after adding graphite powder. NaKC was mixed with fluorinated foam aluminum, ensuring that the foam aluminum was completely immersed in the NaKC liquid alloy. Figure 1c shows that the untreated AF did not have excellent surface affinity with NaKC. However, NaKC alloy was uniformly filled into the pores of the treated AF (noted as NaKC-FAF), and no uneven structure was observed on the surface under macroscopic observation (Figure 1d), which implied that FAF owned excellent wettability with NaKC.

Different electrolytes, namely 1.0 M NaClO_4_/EC-PC-5% FEC and 1.0 M NaPF_6_/Diglyme, were separately dropped onto the NaKC-FAF electrode. From Figure 1e, it can be seen that when the carbonate-based electrolyte is dropped onto the NaKC-FAF electrode, the electrode color remains unchanged and there is no significant change for the liquid sodium–potassium metal inside the electrode. However, when the ether-based electrolyte is applied, as shown in Figure 1f, the part of the electrolyte in contact with the surface of the NaKC-FAF electrode changes from silver to black. Additionally, a small amount of liquid sodium–potassium metal escapes to the electrode surface, forming spherical droplets on the NaKC-FAF electrode (the inserted picture of Figure 1f). A similar phenomenon has been described in the literature [13], which speculates that the slight dissolution of the Na–K in ethers may destroy the physical bonding between the liquid Na–K and the porous material. The NaKC-FAF electrode was found to be more structurally stable in the carbonate-based electrolyte (1.0 M NaClO_4_/EC-PC-5% FEC), and thus, this electrolyte was used throughout subsequent experiments. Additionally, at a current density of 1 mA cm^−2^ in the carbonate-based electrolyte, the Na|Na and NaKC-FAF|NaKC-FAF batteries cycled for 50 h. In Figure 1g,h, the surface of the Na electrode showed powdery substances and uneven color distribution, while the NaKC-FAF electrode still retained a metallic luster, indicating that the surface of the NaKC-FAF electrode was more stable.

**Figure 2:**
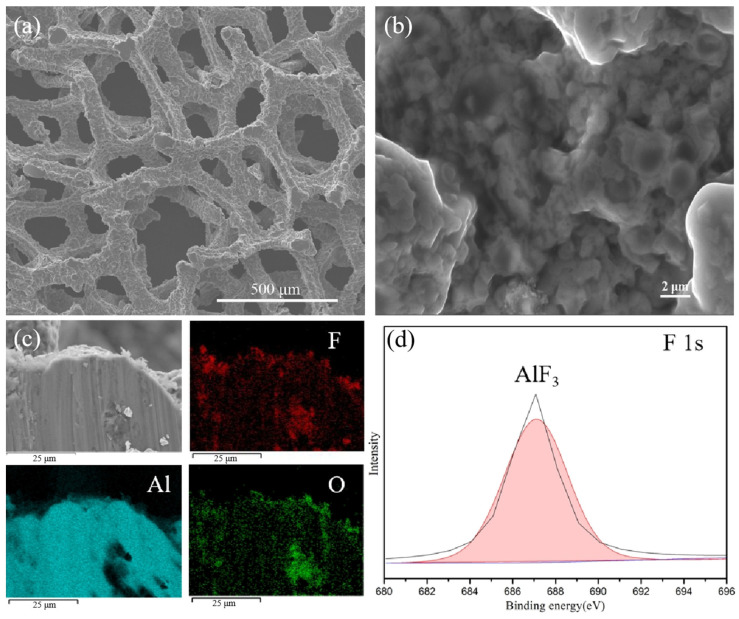
SEM and XPS images of fluorinated aluminum foam. (**a**,**b**) Morphology of fluorinated aluminum foam (FAF), (**c**) element distribution of F, Al and O elements, and (**d**) XPS fitting spectrum of fluorine (F) elements on FAF.

The SEM image of fluorinated aluminum foam (FAF) (Figure 2a,b) reveals that the foam aluminum treated with HF still retains a similar morphology to that of the raw material structure, and there exist some micropores on the surface. Figure S1 also demonstrates the preserved integrity of the fluorinated aluminum foam, with the appearance of a porous interface relative to the original foam aluminum. The elemental overlay results obtained from EDAX illustrate that the fluorine (F) elements are distributed homogeneously on the surface of the porous aluminum foam (Figure 2c). The ingredients analysis carried out via XPS fitting spectrum (shown in Figure 2d) highlights that the F elements exist in the compound of AlF_3_ within the surface of the aluminum foam, and the aluminum element at the interface is mainly composed of Al_2_O_3_ and AlF_3_ (Figure S2)_._ It indicates that the fluorinated treatment was successfully conducted.

**Figure 3:**
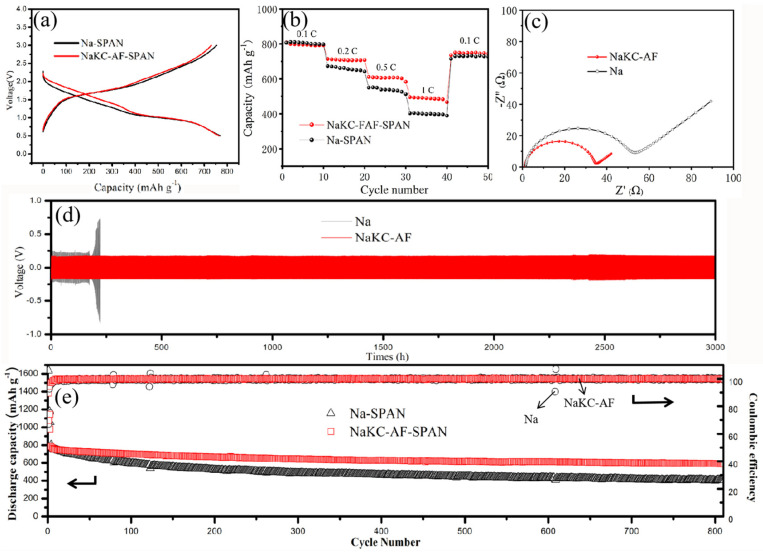
Electrochemical performance of Na and NAKC-FAF cells: (**a**) discharge–charge curves of Na−SPAN and NaKC-FAF−SPAN full cells at 0.2 C, (**b**) rate performance of Na-SPAN and NaKC-FAF−SPAN full cells, (**c**) AC impedance test of Na|Na and NaKC-FAF|NaKC-FAF symmetric cells after cycling, (**d**) cycling stability of Na|Na and NaKC-FAF|NaKC-FAF symmetric cells, and (**e**) long-term cyclic performance of Na-SPAN and NaKC-FAF−SPAN full cells at 0.2 C.

Due to the simple preparation and stable performance of SPAN [14], and the fact that both SPAN and NaKC-FAF are more stable in carbonates, we chose NaKC-FAF−SPAN batteries as the focus of our research. Figure 3a displays the typical cyclic galvanostatic discharge–charge curves of Na-SPAN and NaKC-FAF−SPAN full cells. After capacity normalization during the 20th cycle of the discharge process, it was observed that the voltage plateau of the NaKC-FAF−SPAN battery was approximately 0.2 V higher than that of the Na-SPAN battery, which is consistent with the results in Figure S3. This suggests that the dissolved potassium ions participate in the reaction of the cathode sulfurized polyacrylonitrile (SPAN) during the discharge process. Potassium ions have a lower redox potential (−2.931 V vs. Standard Hydrogen Electrode (SHE)) compared to Na/Na^+^ (−2.71 V vs. SHE), which results in a battery with potassium ions having a higher voltage plateau during discharge [15]. The voltage difference between the two discharge curves being equal to the difference in SHE potentials also confirms the participation of potassium ions in the entire discharge process.

Figure 3b illustrates the cycling performance of the two compared batteries at different current densities. It was evident that the NaKC-AF−SPAN battery (red line) exhibited a better rate performance than the Na-SPAN battery (black line), especially at a high current density of 1.0 C (1 C = 500 mAh g^−1^). This is due to the more stable interface of the NaKC-FAF electrode, and the NaKC-FAF−SPAN battery showed faster kinetics with increasing current densities, producing relatively high capacities at 0.1 C, 0.2 C, 0.5 C and 1.0 C, which were 793 mAh g^−1^, 715 mAh g^−1^, 580 mAh g^−1^ and 501 mAh g^−1^, respectively. In contrast, the capacities of the Na−SPAN battery under the same conditions were lower, which were 798 mAh g^−1^, 642 mAh g^−1^, 538 mAh g^−1^ and 412 mAh g^−1^, respectively. Additionally, the discharge–charge profile during rate performance is shown in Figure S4, and the NaKC-FAF−SPAN battery showed a better rate performance. These results indicate that the NaKC-FAF composite electrode can effectively improve the rapid charge and discharge capability of the battery.

To investigate the cycling stability of the NaKC-FAF electrode, NaKC-FAF|NaKC-FAF symmetric cells were tested and compared with Na|Na symmetric cells. After 150 h of charge/discharge cycling, the symmetric cells underwent AC impedance spectroscopy testing. As shown in Figure 3c, the interface resistance of the NaKC-FAF electrode was much lower than that of the Na electrode in carbonate-based electrolytes. Furthermore, the interface resistance after cycling of the full cell also showed similar conclusion (Figure S5). Additionally, at a current density of 1 mA cm^−2^/1 mAh cm^−2^, the NaKC-FAF|NaKC-FAF battery exhibited stable cycling for over 3000 h at lower polarization voltages (Figure 3d), while the cycling stability of the Na|Na battery was only about 200 h, indicating that the NaKC-FAF|NaKC-FAF electrode has better cycling stability. Moreover, the polarization voltage of the symmetrical battery was used for comparison (Figure S6), in which NaKC-FAF|NaKC-FAF exhibited a smaller polarization voltage.

Figure 3e presents a comparison of the long-term cycling stability of the full batteries. In the first five cycles, the coulombic efficiency was relatively low due to the irreversible consumption of Na^+^/K^+^ by SPAN [16]. During the first 20 cycles, the specific capacities of the Na−SPAN and NaKC-FAF−SPAN batteries were maintained at 750 mAh g^−1^ without significant difference, indicating that Na and NaKC-FAF electrodes had almost no effect on the cycling performance in the initial stage. However, as the cycling continued, the specific capacity of the Na-SPAN battery decreased significantly, while the NaKC-FAF−SPAN battery showed better stability over 800 cycles. The capacity retention of NaKC-FAF−SPAN reached 72%, while that of Na−SPAN was only 49%. This indicates that the NaKC-FAF liquid alloy electrode can effectively improve the cycling performance of sodium–sulfur batteries.

**Figure 4:**
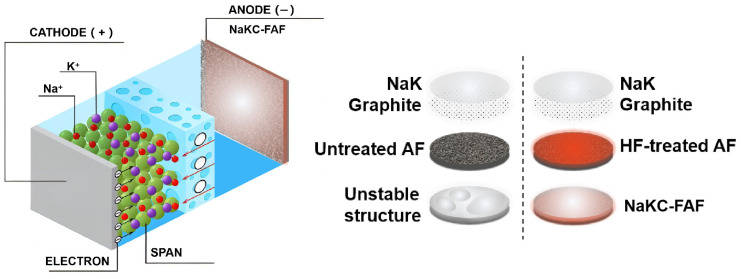
Schematic diagram of NaKC-FAF anode.

Figure 4 shows a schematic diagram of the NaKC-FAF electrode. By adding graphite powder to the NaK liquid alloy, the surface tension of the NaKC alloy can be reduced, while fluorinated foam aluminum exhibits a higher affinity for the NaKC alloy. Therefore, the NaKC alloy can be well combined with fluorinated foam aluminum, allowing the NaKC alloy to be uniformly immobilized in the fluorinated foam aluminum. This provides a fast electron transfer pathway for Na^+^ and K^+^ in the foam aluminum, whereas the non-fluorinated AF electrode has a weaker affinity with NaKC alloy and poorer uniformity after being combined with the sodium–potassium alloy. Additionally, the NaKC composite has a certain fluidity and its interface has self-repairing properties, which can prevent the formation of sodium and potassium dendrites. Therefore, through the synergistic effect between the FAF and the NaKC alloy, the NaKC-FAF composite anode effectively suppresses the formation of dendrites and improves the cycling stability of the electrode interface.

## 4. Conclusions

In summary, we created a novel anode material (NaKC-FAF) by combining sodium–potassium liquid alloy and fluorinated aluminum foam. According to the statistics, the NaKC-FAF symmetrical cell provides a stable circuit exceeding 3000 h in 1.0 M NaClO_4_/EC-PC-5% FEC electrolyte, and a full battery with an NaKC-FAF anode and a SPAN cathode exhibits approximately 72% capacity retention after 800 cycles. It is worth mentioning that aluminum foam is a normal and eco-friendly material with a low density. We believe that this facile and novel design will contribute to the development of next-generation high-energy-density electrochemical systems.

## Data Availability

Data are contained within the article.

## Supplementary Materials

The following supporting information can be downloaded at: https://www.mdpi.com/article/10.3390/ma16237269/s1, Figure S1: The SEM/photographic images of (a) pristine Al foam and (b) FAF; Figure S2: X-ray photoelectron spectroscopy (XPS) spectra of the Al element of FAF electrode; Figure S3: The CV curves of Na-SPAN/NaKC-FAF-SPAN at 0.1 mV s^-1^; Figure S4: The Discharge-Charge profile during the rate performance: (a) Na-SPAN, (b) NaKC-FAF-SPAN; Figure S5: AC impedance test of Na-SPAN and NaKC-FAF-SPAN cells after 500th cycling; Figure S6: The polarization voltage of the symmetrical battery.
