# Supplementary material for: A Novel Sodium–Potassium Anode Supported by Fluorinated Aluminum Foam
Source: Materials (Basel). 2023 Nov 22;16(23):7269. doi: 10.3390/ma16237269 (PMC10707027; doi:10.3390/ma16237269)
Supplement: Supplementary file 1 [file materials-16-07269-s001.zip › materials-2687849-supplementary.pdf]

A Novel Sodium–Potassium Anode Supported by Fluorinated Aluminum Foam

Jin Lou [1,*], Jingan Zhou [2], Xiaosong Ma [2], Kanghua Chen [1] and Songyi Chen [1]

1 Light Alloy Metal Research Institute, Central South University, Changsha 410083, China; khchen@csu.edu.cn (K.C.); sychen08@csu.edu.cn (S.C.)
2 Hunan Provincial Key Laboratory of Carbon Neutrality and Intelligent Energy, School of Resources & Environment, Hunan University of Technology and Business, Changsha 410205, China; 17373564507@163.com (J.Z.); ma15367552578@163.com (X.M.)
* Correspondence: jinlou@csu.edu.cn

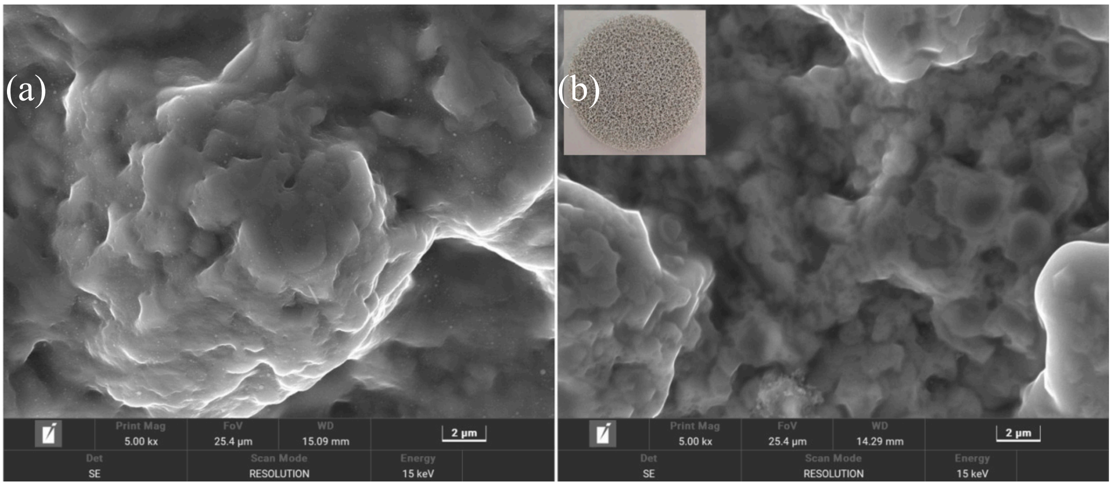

Figure S1. The SEM/photographic images of (a) pristine Al foam and (b) FAF.

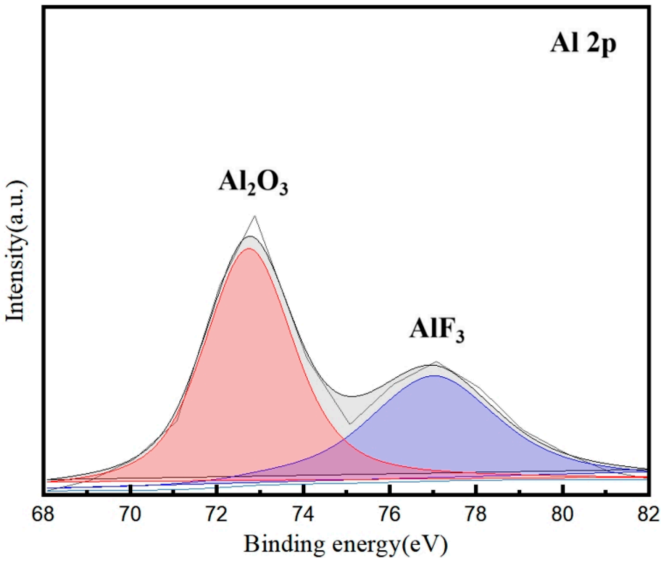

Figure S2. X-ray photoelectron spectroscopy (XPS) spectra of the Al element of FAF electrode.

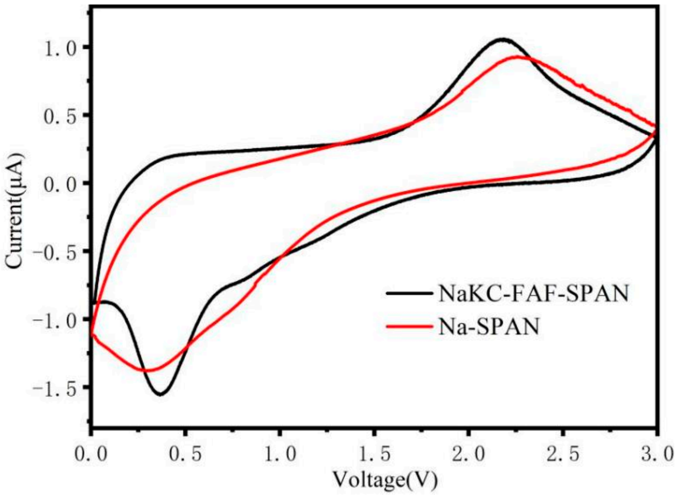

Figure S3. The CV curves of Na-SPAN/NaKC-FAF-SPAN at 0.1 mV s^{-1}

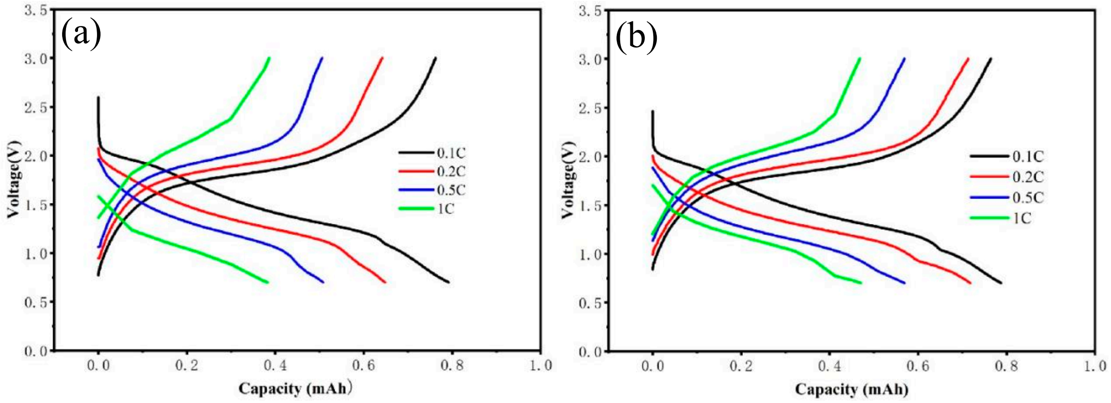

Figure S4. The Discharge-Charge profile during the rate performance: (a) Na-SPAN, (b) NaKC-FAF-SPAN.

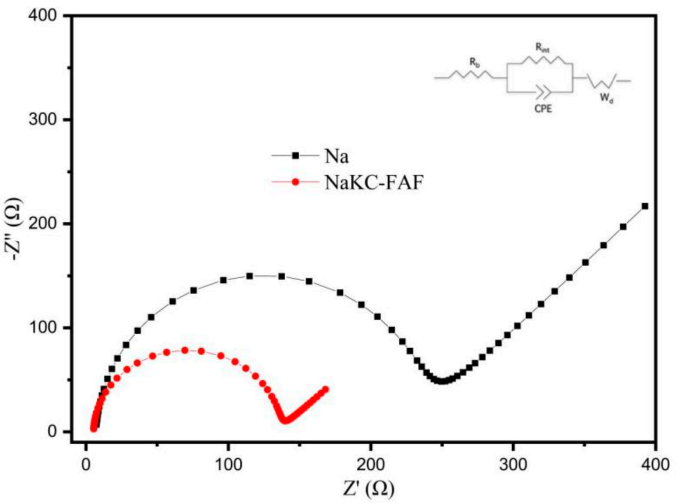

Figure S5. AC impedance test of Na-SPAN and NaKC-FAF-SPAN cells after

500th cycling

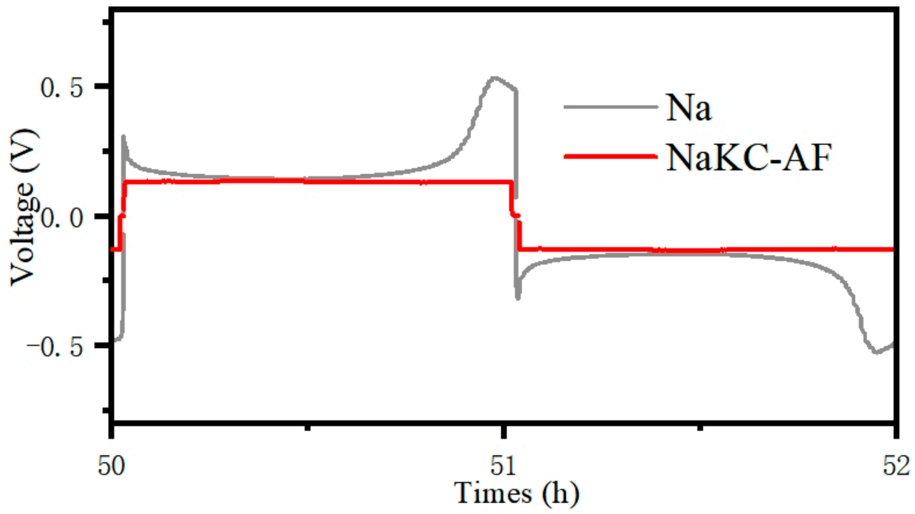

Figure S6. The polarization voltage of the symmetrical battery
